# A Novel Closed-Chest Porcine Model of Chronic Ischemic Heart Failure Suitable for Experimental Research in Cardiovascular Disease

**DOI:** 10.1155/2013/410631

**Published:** 2013-09-15

**Authors:** Giuseppe Biondi-Zoccai, Elena De Falco, Mariangela Peruzzi, Elena Cavarretta, Massimo Mancone, Omar Leoni, Maria Emiliana Caristo, Marzia Lotrionte, Antonino G. M. Marullo, Antonio Amodeo, Luca Pacini, Antonella Calogero, Vincenzo Petrozza, Isotta Chimenti, Fabrizio D'Ascenzo, Giacomo Frati

**Affiliations:** ^1^Department of Medico-Surgical Sciences and Biotechnologies, Sapienza University of Rome, Corso della Repubblica 79, 04100 Latina, Italy; ^2^Department of Cardiovascular, Respiratory, Nephrologic, Anesthesiologic and Geriatric Sciences, Sapienza University of Rome, Viale del Policlinico 155, 00161 Rome, Italy; ^3^Center of Experimental Research, Catholic University of Rome, Largo Agostino Gemelli 8, 00168 Rome, Italy; ^4^Division of Cardiology, Catholic University of Rome, Largo Agostino Gemelli 8, 00168 Rome, Italy; ^5^Department of Cardiac Surgery, Bambino Gesù Hospital, Piazza di Sant'Onofrio 4, 00165 Rome, Italy; ^6^Division of Cardiology, University of Turin, Corso Bramante 88-90, 10126 Turin, Italy; ^7^IRCCS Neuromed, Via Atinense 18, 86077 Pozzilli, Italy

## Abstract

Cardiac pathologies are among the leading causes of mortality and morbidity in industrialized countries, with myocardial infarction (MI) representing one of the major conditions leading to heart failure (HF). Hitherto, the development of consistent, stable, and reproducible models of closed-chest MI in large animals, meeting the clinical realism of a patient with HF subsequent to chronic ischemic necrosis, has not been successful. We hereby report the design and ensuing application of a novel porcine experimental model of closed-chest chronic ischemia suitable for biomedical research, mimicking post-MI HF. We also emphasize the key procedural steps involved in replicating this unprecedented model, from femoral artery and vein catheterization to MI induction by permanent occlusion of the left anterior descending coronary artery through superselective deployment of platinum-nylon coils, as well as endomyocardial biopsy sampling for histologic analysis and cell harvesting. Our model could indeed represent a valuable contribution and tool for translational research, providing precious insights to understand and overcome the many hurdles concerning, and currently quenching, the preclinical steps mandatory for the clinical translation of new cardiovascular technologies for personalized HF treatments.

## 1. Introduction

Cardiac pathologies are among the leading causes of mortality and morbidity in industrialized countries, with heart failure (HF) representing the final common pathway for many diseases that affect the heart and defining a syndrome characterized by inadequate performance of the heart that negatively affects whole body blood supply [1]. Myocardial infarction (MI) is one of the major conditions leading to HF, having an ominous impact on public health in terms of mortality and morbidity [2]. The hemodynamic overload generated by MI imposes mechanical and neurohumoral modifications on cardiac walls, triggering complex biological responses that culminate in tissue remodeling. This response initially starts as compensatory left ventricular hypertrophy but eventually evolves towards maladaptive remodeling, possibly triggering transition to HF. The cascade of events that begins with cardiac hypertrophy, attempting to set on a compensatory response and finally leading to HF, is characterized by contractile dysfunction and cell death of stressed cardiomyocytes, reduced capillary density, inflammation and fibrosis [3]. To date available medical treatments aim more at preserving residual, albeit compromised, cardiac function rather than at restoring lost functions. Besides, available drug therapies act by decreasing cardiac workload by reducing heart rate and blood pressure (such as *β*-blockers), preserving blood flow in coronaries (such as nitrates), and by blocking or reversing the remodeling process (such as angiotensin-converting enzyme (ACE) inhibitors), while not addressing the specific issue of recovering the loss of function due to massive muscular death. Even cardiac surgery remains a palliative management, not always suitable for catastrophic events like large myocardial damage due to huge infarction and cell demise.

However, all research approaches focusing on the improvement of cardiac function by cell therapy have hitherto encountered only incomplete success and generated conflicting results with no clear evidence of heart regeneration potential, which is mainly due to unsolved issues related to low survival and engraftment rate of injected cells, as well as the occurrence of complications such as inflammation or fibrosis [4]. In that sense, the scientific community has now to take a step back as the clinical outcome highlighted by the most recent clinical trials has only partially mirrored the expected results based on preclinical animal models, in terms of actual engraftment, survival, differentiation, and functional recovery. 

Thus, the development of a consistent, stable, and reproducible model of closed-chest MI and cell delivery is mandatory as an efficient and realistic tool for the preclinical evaluation of cell therapy procedures. Nowadays, in the scholarly literature, several in vivo animal models reproducing HF are available as a result of genetic modifications, surgical ligature of the coronary arteries (with or without reperfusion), microembolization, cryoinjuries to the epicardium, electrical stimulation at a rapid pace, modifications of load, or toxic pharmacological treatments [5–10]. All these models have certainly allowed us to improve our mechanistic knowledge, but they do not go far enough in meeting the clinical reality of a patient with HF subsequent to chronic ischemia. Indeed, small animal models such as rodents have provided significant insights into human cardiac pathophysiology. Specifically, rodent and human hearts are greatly different in their dimension, structure, heart rate, oxygen consumption, regional and global contractility, protein expression, and even in resident stem cell populations [11], with the consequent and clear need for models of HF in large animals. The emergence of such large animal models in cardiovascular research fields such as MI, HF, valvular disease, heart transplantation, and ventricular assist devices (VAD) reflects the close similarity of these animals to human anatomy and physiology. The size of pigs (e.g., female Landrace pigs, weighing 30–35 kg, aged 3–12 months) allows the use of surgical equipment and imaging modalities similar to those used in humans, aiming at reproducing a real clinical situation with the employment of human-sized instruments while enabling the development and application of a unique model close to the clinical reality of a patient with HF.

In the light of this, our aim was to devise and apply a novel porcine closed-chest experimental model for biomedical research, describing how to perform a full procedure from femoral artery and vein catheterization to MI creation by minimally invasive transcatheter permanent coronary occlusion at the level of the left anterior descending artery (LAD) with selective deployment of intraluminal coils. We also describe transcatheter endomyocardial bioptic sample collection in order to isolate, characterize, and expand autologous resident cardiac progenitor cells [12], representing the basis for the clinical translation of new possible protocols for regenerative medicine. Results from this report aim at proposing a consistent, stable, reproducible, and, most importantly, clinically relevant model of closed-chest MI and ensuing HF, resembling more realistically the medical history of a patient with this condition, and thus representing a more accurate pathophysiological model to test possible cardiac progenitor cell-based technologies for personalized HF treatments closer to clinical translation. 

## 2. Materials and Methods

### 2.1. In Vivo Experimental Protocol

All animals were handled in compliance with the European Convention on Animal Care and received humane care in accordance with the Principles of Laboratory Animal Care and the Guide for the Care and Use of Laboratory Animals. The experimental protocol was approved by local authorities and by the bioethical committee of the Catholic University, Rome, Italy (Protocol CESA/P/52/2012-13/12/2012).

### 2.2. Animal Preparation and Anesthesia

Experimental preparation and surgical protocol were performed under sterile conditions. Atropine (0.02 mg/kg IM), ketamine (15 mg/kg IM), and diazepam (0.1 mg/kg IM) were used for premedication, and intravenous access was obtained with a 21- or 22-Gauge needle in the ear vein. Anesthesia was induced by IV injection of ketamine (35 mg/kg) and diazepam (0.1 mg/kg). All animals were intubated with an endotracheal tube of 5 to 7 mm internal diameter, and general anesthesia was maintained with 1% to 2% isoflurane supplemented with oxygen, both supplied by an Aliseo mechanical ventilator (Datex Ohmeda, General Electric, Fairfield, CN, USA) and a constant rate IV infusion of propofol at 6 mg/kg/h for the first two hours and then at 4 mg/kg. Muscle relaxation was obtained with a constant rate IV infusion of atracurium besylate (0.5 mg/kg). Ventilator parameters were set according to each animal body weight (tidal volume 10 mL/kg, respiratory rate 16/minute, I/E 1 : 2, FiO2 40%, heart rate 70/130 bpm, SBP 110–120/70–80 mm Hg, central venous pressure 4–10 mm Hg). During the anesthesia we used a IV infusion of Ringer Lactate crystalloid solution of 10 mL/kg. In order to prevent infection, we administered enrofloxacin (5 mg/kg qd IM for 5 days), whereas for the intra- and postoperatory pain relief we used tramadol at 2/4 mg/kg bid IM/EV for the first 48 h, followed by ketoprofen: 2 mg/kg qd IM for further 3 days. Before starting all the procedures (about 30 minutes prior to the intervention) an IV infusion of 1.0–2.5 mg/kg amiodarone was started in order to prevent possible ventricular arrhythmias, and 2000 IU heparin IV was administered in order to avoid thromboembolic phenomena. 

### 2.3. Ventriculography, Bioptic Sampling, and Coil Deployment

**Figure 1:**
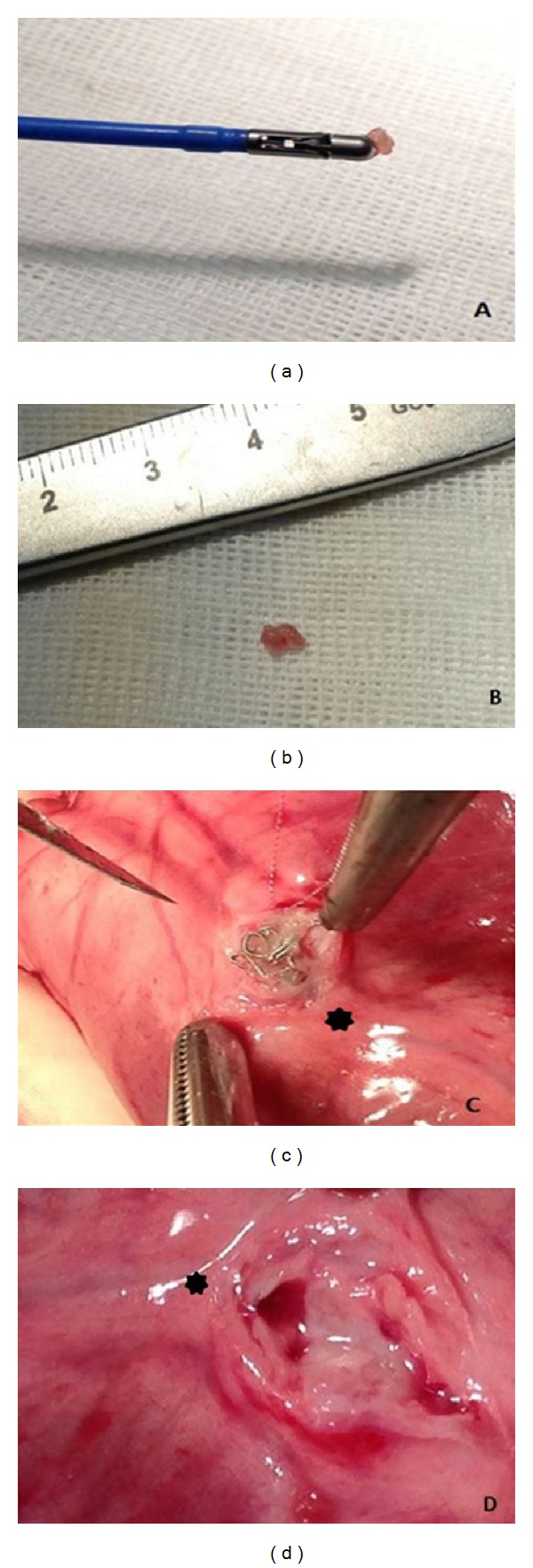
((a) and (b)) Ventricular biopsies obtained using a standard clinical cardiac bioptome introduced through a 7-French sheath. (c) Evidence of the intraluminal platinum-nylon coil (Axium, Covidien, Mansfield, MA, USA) at 1 month followup (distal left anterior descending (LAD) represented by the star). (d) Evidence of the coronary lumen at 1 month highlighting the segment of the coronary vessel distal to the occluded segment (distal LAD represented by the star).

**Figure 2:**
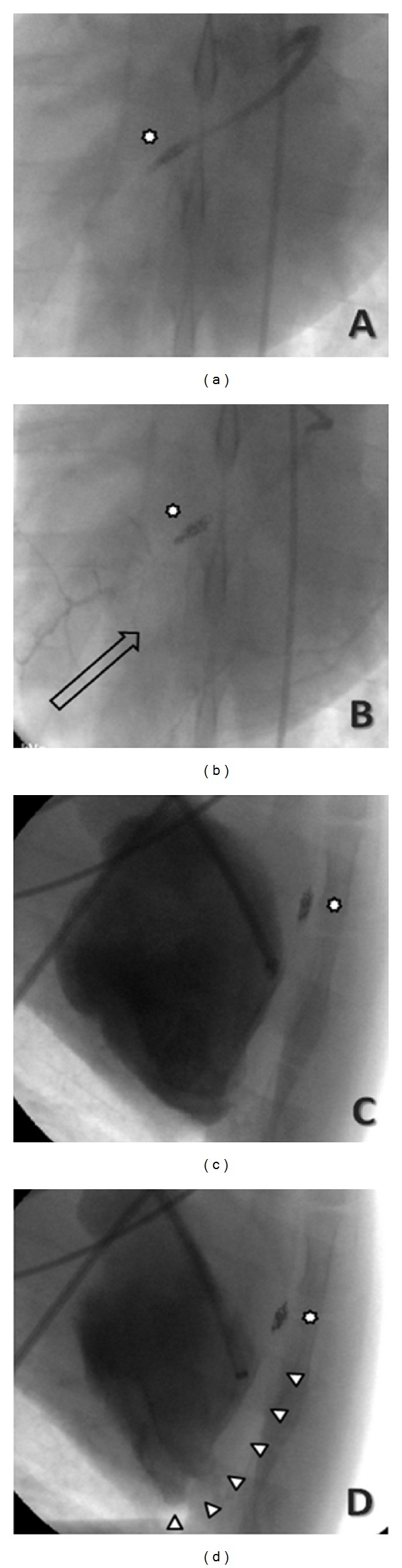
Coronary angiography of the occlusion of the mid tract of the left anterior descending coronary leading to myocardial infarction after deploying an intraluminal platinum-nylon coil (Axium, Covidien, Mansfield, MA, USA) at (a) 1 month followup (star represents the coil), with retrograde collateral flow from the left circumflex (b). Follow-up ventriculography showing the diastolic (c) and systolic (d) phases. Arrowheads represent the akinetic segments.

The right femoral artery was willingly punctured in all animals according to Seldinger and cannulated with a short 5-French sheath exchanged for a 90 cm 7-French sheath (Cordis, Miami, FL, USA). Left ventriculography was performed in a right anterior oblique projection, with injection of dye through either a 5-French pigtail catheter (Boston Scientific, Natick, MA, USA), a 5-French Amplatz Right 1 diagnostic catheter (Boston Scientific), deployed by means of a 300 cm 0.035′′ angiographic guide wire (Boston Scientific), or the 7-French sheath. Then, endomyocardial biopsy was performed aiming at the interventricular septum with a 7-French device (BiPal, Cordis). After obtaining a suitable number of endomyocardial samples (5 to 7 per animal, each weighing from 10 to 50 mg, see Figure 1), the bioptome was exchanged for an Amplatz Right 1 or Amplatz Left 1 Judkins Right guiding catheters, which were used to cannulate the left coronary artery for selective coronary arteriography and coil deployment. A 0.014′′ J-tipped floppy angioplasty guide wire (ChoICE PT floppy, Boston Scientific) was then inserted into the LAD under fluoroscopic guidance, and coronary occlusion was achieved by deploying one or more 4.0 × 10 mm platinum-nylon microcoils (Axium, Covidien, Mansfield, MA, US) distally to the ostium of the first major diagonal branch. To further ensure that the occlusion was permanent, 20 mg of protamine was administered IV after deployment of the coil. Complete cessation of flow into the distal LAD was then confirmed by angiography in all cases (Figure 2). Amiodarone was discontinued 30 minutes after the evidence of ischemia, even if only one case of significant arrhythmias occurred. After all the procedures animals were gradually weaned from anesthesia and allowed to recover in specific single cages. For postoperatory pain relief we used tramadol at 2/4 mg/kg bid IM/IV for the first 48 hours, followed by ketoprofen: 2 mg/kg qd IM for further 3 days. All animals were monitored (4 times/day) and handled in compliance with the European Convention on Animal Care and received humane care in accordance with the Principles of Laboratory Animal Care and the Guide for the Care and Use of Laboratory Animals.

### 2.4. Electrocardiography and Echocardiography

To assess for signs of acute myocardial injury, continuous monitoring by electrocardiography (ECG) was performed. Changes in 12-lead ECG were also appraised to confirm ongoing MI, recording at 25 mm/s, 40 Hz, and 10 mm/mV in all anesthetized and immobilized animals in a supine position. Catheters and sheaths were removed, with hemostasis achieved with manual compression, while transthoracic echocardiography (1 hour after-procedure) was performed to evaluate wall motion abnormalities, confirming in real time the presence of hypokinesia/akinesia of the anterior-lateral wall. Echocardiographic evaluation was performed at baseline, 1 hour after the occlusion, and then at 1-week and 1-month followup. Animals were investigated both in a right and left lateral positions. An experienced cardiologist performed all echocardiographic studies. A commercially available CX-50 machine (Philips, Andover, MA, USA), equipped with a 1–5 MHz probe (S5-1 PureWave sector array), was used for all examinations. Two-dimensional and M-mode data were acquired in parasternal long- and short-axis views at the level of papillary muscles and in apical four-chamber view. At least three consecutive beats were acquired and digitally recorded for off-line analysis performed by two experienced cardiologists with proprietary software (Philips). Left ventricular (LV) diameters, thicknesses, ejection fraction (LVEF), fractional shortening (LVFS), and LV volumes were obtained. The euthanasia was obtained after 30 days with the administration of Tanax (0.3 mL/kg IV; Intervet Italia, Segrate, Italy). 

Hearts were explanted, rinsed 8–10 times with saline solution for a total of 5 minutes, prepared by surgical opening of the right and left atria and immediately submerged in a medical grade formaldehyde-buffered solution in sterile conditions for histological and immunohistochemical assessment. 

### 2.5. Tissue Processing and Histology

After sacrifice, hearts were harvested and fixed with formaldehyde 4% (Kaltek, Padua, Italy) for 48 hours. The presence and the location of the infarct area were assessed by two expert clinical pathologists through macroscopic examination. Paraffin sections (2 *μ*m) were then obtained from fixed hearts (PM2255 microtome, Leica, Solms, Germany) and stained with haematoxylin/eosin in the automated station St 5020 (Leica) to microscopically analyse the myocardial tissue. Subsequently, tissue sections were stained with Masson's Trichrome (Menarini, Parma, Italy, cat. N° 04010802), and images were acquired by a DSIGHT Fluo microscope (Menarini).

## 3. Results

### 3.1. Functional Evaluation

One out of 5 animals (20%) showed ventricular fibrillation immediately after permanent LAD occlusion, and in this case resuscitation was achieved with repeated adrenaline boluses (10 mcg/kg IV) and a single biphasic DC shock at 200 Joules. Heart rates were detected at baseline (79.7 ± 3.5 bpm), soon after the occlusion (100.1 ± 7.3 bpm) and during all the phases of chronic HF subsequent to permanent coronary occlusion (85.3 ± 5.9 bpm). ECG was performed at baseline, continuously during the intervention (pre-, intra-, and postcoronary occlusion), at 1 week and at the end of the experiment confirming in all significant ST-segment elevation in the acute phase and a necrosis pattern afterwards.

**Figure 3:**
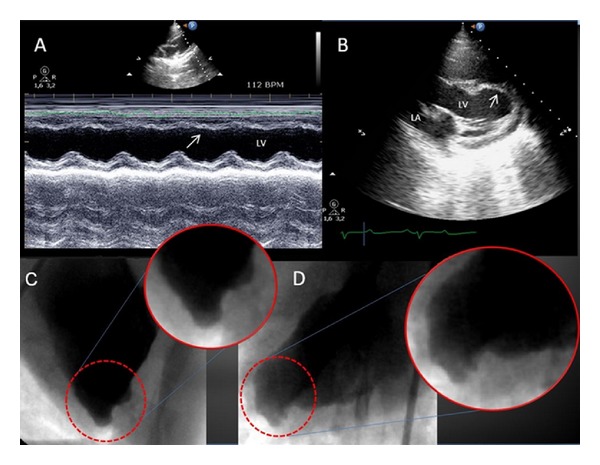
(A) Echocardiography at 1 month: M-mode image of left ventricle (LV); the arrow points at the anterior interventricular septum, which appears severely hypokinetic/akinetic, in comparison with the posterior wall. (B) Two-dimensional parasternal long axis; the LV apex appears remodeled and aneurysmatic (arrow), and wall thickness is markedly reduced in comparison with the basal segment. LA: left atrium. Follow-up ventriculography (1 month), with magnified views, showing a remodeled and aneurysmatic LV apex, in the right oblique (C) and anteroposterior (D) views.

Coronary occlusion with the subsequent MI resulted in immediate reduction in LVEF and LVFS, from 62.7 ± 2.3% to 45.3 ± 5.1% and from 33.6 ± 1.6% to 22.1 ± 2.9%, respectively. Ongoing remodeling of the LV was evident by continued reduction in LVEF and LVFS over the following 4 weeks (from 45.3 ± 5.1% to 35.9 ± 3.2% and from 22.1 ± 2.9% to 18.2 ± 4.3%, resp.,) and by an increase in LV end-diastolic diameters and volumes (Table 1). M-mode imaging of left ventricle showed a severely hypokinetic/akinetic anterior interventricular septum, in comparison with the posterior wall (Figure 3). Two-dimensional parasternal long-axis view evidenced an aneurysmatic left ventricular apex, with the distal and median wall thicknesses markedly reduced in comparison with the basal segment that resulted normokinetic (Figure 3). This seems to mirror the LV remodeling observed in humans after a MI.

Coronary angiography (Figure 2) performed at 1 month highlighted the completeocclusion of the mid tract of the LAD consequent to the deployment of the intraluminal platinum-nylon coil. In two out of five animals (20%), selective coronary angiography of the LAD at 1-month followup showed a faint retrograde collateral flow from the left circumflex (Figure 2). Follow-up ventriculography performed at 1 month confirmed the presence of akinetic segments corresponding to the lateral wall of LV and a remodeled and aneurysmatic apex (Figures 2 and 3). 

Mortality in this series was 0% likely in reason of the fact that, after the set-up stage, we always deployed the platinum-nylon microcoils distally to the ostium of the first major diagonal branch. Indeed, deployment of the microcoil proximally to the ostium of the first major diagonal branch resulted in immediate ventricular fibrillation unresponsive to pharmacological and/or electrical treatment, in keeping with prior experiences with very proximal LAD balloon occlusion [9].

### 3.2. Histological Analysis

**Figure 4:**
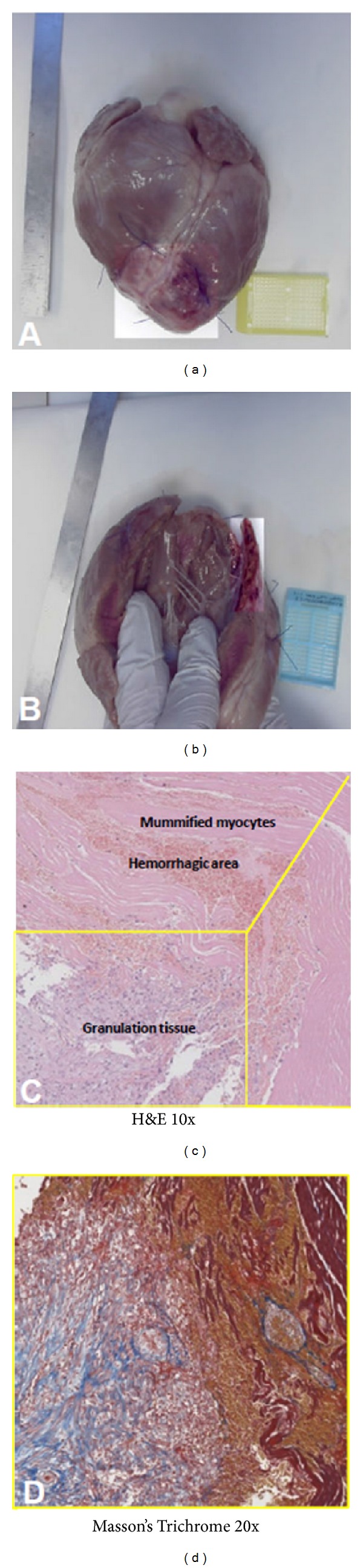
(a) Macroscopic analysis of the porcine heart showing the infarcted area, which appears brighter than the rest of the myocardium. (b) Ventricular wall appears thinned in the area of transmural infarction. (c) Hematoxylin/eosin (H&E) staining of the porcine model showing the haemorrhagic and the necrotic zones in the infarcted area. Infiltration of granulation tissue and fibroblastic/macrophagic cells is also observed (10x magnification). (d) Masson's Trichrome staining on a selected area of the section confirming both fibrotic connective and granulation tissues (20x magnification).

All hearts showed transmural scar and massive fibrosis consistent with acute coronary occlusion due to thrombosis [13]. The infarct area was white, shrunken, thin, and firm. The average of total infarct area corresponds to 31.8 ± 1.5 cm^2^ (Figure 4). Healed infarcts displayed a white scar surrounded by small areas with congestion and vasodilatation. The ventricular wall was thinned as it appears in transmural infarction. Indeed the infarcts occupied more than 60% of the LV wall, from the subendocardial to the epicardial surface. Microscopic findings demonstrate the presence of granulation tissue with a large number of macrophages often engulfed with debris of the necrotic myocytes and hemosiderin. Fibroblasts actively producing collagen were found in the area of healing as well as angiogenesis. Rare eosinophils were also present within the chronic inflammatory areas. The central area of infarction showed a small unhealed zone with mummified myocytes and limited necrosis. Hemorrhagic areas were also present. Masson's Trichrome staining confirmed the presence of fibrotic area evenly distributed in the tissue sections. Representative images of histology analysis are displayed in Figure 4.

## 4. Discussion

In the last two decades, rapid advances have been made in understanding genetic, molecular, and pathophysiological pathways involved in the development and homeostasis of the mammalian heart, as well as perturbations that may influence cardiac physiology. Despite all this, to date reliable animal models, both suitable for research purposes and closely resembling human heart failure, still do not exist. The importance to develop such models is based on major limitations that occur particularly with murine in vivo models, which show relevant physiological differences with human hearts [11], although very useful and less expensive than large animals.

Among large animals, pigs are certainly preferred, due to the collateral coronary circulation and arterial anatomy very similar to humans and most importantly because it is even possible to predict the infarct size [14–16]. Moreover, animal models of HF, as opposed to isolated cells or organs, permit a more accurate, realistic, and complete analysis of the physiological effects of cardiac dysfunction, which are of great importance in the overall HF epiphenomenon. 

In this study we have attempted to address the key issue of creating an animal model of HF which could simplify an extremely complex and challenging syndrome into manageable cardiovascular research questions. Specifically, the major novelty of our model lies in the fact that for the first time we propose a chronic post-MI HF model with a real LV remodeling, based on a closed-chest technique [17], combined with the permanent occlusion of the LAD. This model has been conceived to allow the possibility of safely performing afterward a surgical operation such as a VAD implantation or a stem cells delivery without the jeopardy of a redo procedure, consequently avoiding all related complications such as surgical adherences, bleeding, and prolonged surgical times. The present model, once optimized the coil deployment site, seems also to have the advantage of dramatically reduced mortality (nihil in our current experience), possibly reducing also the overall cost of research. The validation of our model has been confirmed by the histological analysis, showing pathological features of a 4-week-old MI, very similar to nonreperfused or delayed-reperfused MI (6 to 12 hours). Accordingly, other models exploiting a transient coronary occlusion followed by reperfusion (opposed to our model of a permanent coronary occlusion) do not reflect the current clinical practice, in reason of the fact that the vast majority of patients with acute MI does not receive a reperfusion therapy before 3 to 4 hours from symptoms onset or does not receive it at all [9]. Therefore, this technique might help to more easily mimic the pathophysiology of nonreperfused or delayed-reperfused MI.

In the future, we envision exploiting further this model and evaluating whether it can also prove useful and suitable for cell therapy applications, by obtaining and injecting autologous cardiac resident stem cells and by implantation of left VAD (LVAD) without the challenges of a redo procedure. All research approaches focusing on the improvement of cardiac function by cell therapy have hitherto encountered only incomplete success and generated conflicting results with no clear evidence of heart regeneration potential, which is mainly due to unsolved issues related to low survival and engraftment rate of injected cells, as well as the occurrence of complications such as inflammation or fibrosis [4, 18–20]. In that sense, the scientific community has now to take a step back as the clinical outcome highlighted by the most recent clinical trials has only partially mirrored the expected results based on preclinical animal models, in terms of actual engraftment, survival, differentiation, and functional recovery. Thus, the development of a consistent, stable, and reproducible model of closed-chest MI and biopsy collection is mandatory as a realistic tool for the preclinical evaluation of cell therapy procedures.

Despite the obvious impossibility of selecting one single swine model as the best fitting or performing for all kinds of research needs, nevertheless compared to current surgical in vivo models, our approach could represent a new promising tool for a more realistic clinical translation of novel regenerative medicine technologies. 

This work has several strengths but also some key limitations. The limited sample size, performance by a team of highly skilled clinician investigators, veterinarians, and pathologists, all thoroughly experienced in animal experimental research spanning from small and large sizes, and use of sophisticated equipments and devices may limit the external validity of our results. However, we are confident that applying this model in a similar setting by similarly experienced and skilled operators will yield similarly satisfactory and precise results. 

## 5. Conclusion

It is clear that an intriguing question concerns the choice of the animal model balancing researcher's options between convenience, cost, physiological applicability, and real correspondence to the original human model. We are also aware that a unique animal model cannot exist. Despite all these limitations, large animals still remain the best tool to investigate severe diseases such as cardiovascular diseases.

## Figures and Tables

**Table 1:** Basal and postacute myocardial infarction echocardiographic data.

Echocardiographic data	Basal	Post-AMI 1 hour	Post-AMI 1 week	Post-AMI 1 month
LV end-diastolic diameter (mm)	41.6 ± 1.7	40.8 ± 1.9	42.8 ± 1.2	46.7 ± 1.3
LV end-systolic diameter (mm)	27.6 ± 1.2	31.7 ± 0.8	33.7 ± 3.3	38.3 ± 1.3
LV end-diastolic volume (mL)	77.1 ± 7.2	73.5 ± 8.2	84.3 ± 8.3	99.9 ± 4.6
LV end-systolic volume (mL)	28.7 ± 3.6	40.2 ± 2.5	47.3 ± 2.6	63.2 ± 5.1
LV ejection fraction (%)	62.7 ± 2.3	45.3 ± 5.1	42.8 ± 1.7	35.9 ± 3.2
LV fractional shortening (%)	33.6 ± 1.6	22.1 ± 2.9	21.2 ± 6.9	18.2 ± 4.3

LV: left ventricle.
